# Geospatial Analysis of Access to Emergency Cesarean Delivery for Military and Civilian Populations in the US

**DOI:** 10.1001/jamanetworkopen.2021.42835

**Published:** 2022-01-10

**Authors:** Tarsicio Uribe-Leitz, Bridget Matsas, Michael K. Dalton, Monica A. Lutgendorf, Esther Moberg, Andrew J. Schoenfeld, Eric Goralnick, Joel S. Weissman, Lynette Hamlin, Zara Cooper, Tracey P. Koehlmoos, Molly P. Jarman

**Affiliations:** 1 Center for Surgery and Public Health, Department of Surgery, Brigham and Women’s Hospital, Boston, Massachusetts; 2 Program in Global Surgery and Social Change, Harvard Medical School, Boston, Massachusetts; 3 Department of Sport and Health Sciences, Technical University of Munich, Munich, Germany; 4 Harvard Medical School, Boston, Massachusetts; 5 Division of Maternal-Fetal Medicine, Naval Medical Center San Diego, San Diego, California; 6 Center for Health Services Research, Uniformed Services University of the Health Sciences, Bethesda, Maryland

## Abstract

**Importance:**

Many women in the US, particularly those living in rural areas, have limited access to obstetric care. Military-civilian partnership could improve access to obstetric care and benefit military personnel, their civilian dependents, and the civilian population as a whole.

**Objective:**

To identify medical facilities within military and civilian geographic areas that present opportunities for military-civilian partnership in obstetric care and to assess whether civilian use of military medical treatment facilities (MTFs) could improve access to emergency cesarean delivery care in the US.

**Design, Setting, and Participants:**

This geospatial epidemiological population-based cross-sectional study was conducted from November 2020 to March 2021. ArcGIS Pro software, version 2.7 (Esri), was used to assess population coverage for TRICARE (military insurance) beneficiaries and civilian populations and to estimate 30-minute travel time to 2392 total military and civilian medical facilities that were capable of providing emergency cesarean delivery care in the continental US. Data on health insurance coverage for TRICARE beneficiaries and their civilian dependents per county were obtained from the American Community Survey tables available through ArcGIS Pro software. Demographic characteristics of the general population were obtained from the 2020 key demographic indicators published by Esri. Race and ethnicity were not examined because the data used for this study were aggregated and did not include further categorization by race or ethnicity.

**Main Outcomes and Measures:**

Population coverage rates (measured in percentages) within 30-minute catchment areas, defined as areas that were within a 30-minute travel time to a medical facility capable of providing emergency cesarean delivery care.

**Results:**

A total of 29 MTFs and 2363 civilian hospitals capable of providing emergency cesarean delivery were identified across the contiguous US. Overall, an estimated 167 759 762 women (3 640 000 TRICARE beneficiaries and 164 119 762 civilians) were included in these service areas. The analysis identified 17 of 29 MTFs (58.6%) capable of providing emergency cesarean delivery care that were located within 30-minute catchment areas. Of those, 3 MTFs were the only facilities capable of providing emergency cesarean delivery care within a 30-minute travel time in those regions, and 14 additional MTFs had catchment areas partially overlapping with civilian hospitals that also covered areas without alternative access to emergency cesarean delivery. Expanded use of these 14 MTFs could enhance access to emergency cesarean delivery care not otherwise covered by current civilian hospitals.

**Conclusions and Relevance:**

In this study, 58.6% of MTFs capable of providing emergency cesarean delivery care were located in areas with the potential to improve access to obstetric care within a 30-minute travel time. Maintenance of MTFs in these important access regions could be prioritized in the context of restructuring MTFs. This prioritization has the potential to improve access to emergency cesarean delivery care for underserved civilian populations in the US, particularly among those living in rural areas.

## Key Points

**Question:**

Could military medical treatment facilities (MTFs) improve access to equitable emergency cesarean delivery care for civilian populations in the US?

**Findings:**

In this cross-sectional study of 29 MTFs and 2363 civilian hospitals potentially serving 167 759 762 female TRICARE (military insurance) beneficiaries and civilians, 3 MTFs were identified as the only facilities capable of providing emergency cesarean delivery care within a 30-minute travel time in those regions, and 14 additional MTFs were identified that could improve access to emergency cesarean delivery care not otherwise covered by current civilian hospitals.

**Meaning:**

This study suggests that expanded use of MTFs could improve access to high-quality emergency cesarean delivery care in underserved regions of the US while also supporting military readiness.

## Teaser

This cross-sectional study uses geospatial analysis to identify medical facilities that present opportunities for military-civilian partnership in obstetric care and assess whether expanded use of military medical treatment facilities could improve access to emergency cesarean delivery care.

## Introduction

More than 5 million women in the US live in 1085 of 3007 counties (36%) that do not have available obstetric care or obstetric clinicians (termed maternity care deserts), and an additional 10 million women live in counties with limited access to maternity care, defined as access to facilities, health care professionals, and insurance.^1^ Geospatial analyses of obstetric care within the US reveal limited access to obstetric intensive care units (ICUs) for a substantial portion of the population.^2,3^ Although 87% of women in the US live within 50 miles^4^ of a facility providing level 3 obstetric care (ie, care for complex maternal and fetal conditions and complications) and neonatal intensive care,^5^ only 61.6% of the population has timely emergency access (ie, within 30 minutes) to obstetric care, with even fewer having access to level 3 obstetric and neonatal care within 30 minutes.^2^ Longer travel times to obtain obstetric care have been associated with worse perinatal outcomes, especially when there is a delay in the receipt of emergency cesarean delivery services.^6^

Previous reports have highlighted gaps in obstetric coverage for patients in both the civilian and military health care systems.^2,7,8^ The civilian health care system has substantial obstetric care disparities, with many women experiencing limited access to care, particularly in rural areas. Although the American College of Obstetricians and Gynecologists has provided guidance to more effectively regionalize maternal care and improve access,^7^ ongoing discussions of optimal staffing (ie, right-sizing) within the Military Health System (MHS) have the potential to reduce access to maternal care for military service members and their families owing to the closure or consolidation of military medical treatment facilities (MTFs) offering maternal care.^9,10^ As a result, the Government Accountability Office has recommended that the MHS examine the capabilities of civilian hospitals that surround MTFs before making major changes.^11^

Collaboration between military and civilian health care professionals has been a catalyst for medical innovation since the American Revolution.^12^ In trauma care, military-civilian partnerships have allowed civilian surgeons to incorporate wartime advancements into their practices, and military surgeons have been able to maintain their surgical skills during military drawdowns and peacetimes.^13,14,15,16,17,18,19,20,21^ Existing collaborations (such as those at major trauma centers in Baltimore, Maryland; Cincinnati, Ohio; Jacksonville, Florida; San Antonio, Texas; and Miami, Florida) provide successful models for such partnerships.^17,19,22,23,24^ However, there are opportunities to extend military-civilian collaborations beyond trauma care while addressing population health care needs in the US. One such opportunity includes the delivery of obstetric care, which represents an important area of need in the US civilian health care system and is also the largest service line within the MHS. In this context, it is important to examine how a successful partnership between the MHS and civilian hospitals could improve access to obstetric care and how this partnership would benefit military personnel, their civilian dependents, and the civilian population as a whole. This cross-sectional study sought to identify facilities within military and civilian geographic catchment areas that presented an opportunity for partnerships aimed at improving access to high-quality obstetric care, including emergency cesarean delivery capabilities. Military-civilian partnerships may improve access to cesarean delivery care, supporting the dual MHS aims of ensuring the clinical readiness of the military medical force and the medical readiness of the military force as a whole, particularly among service members living in rural communities.^25^

## Methods

### Study Design

This geospatial epidemiological population-based cross-sectional study was conducted from November 2020 to March 2021. The study assessed population coverage for female TRICARE beneficiaries (TRICARE functions as the health insurance program for the MHS) and civilians and estimated 30-minute travel time to 2392 total military and civilian medical facilities capable of providing emergency cesarean delivery care in the continental US. The study followed the Strengthening the Reporting of Observational Studies in Epidemiology (STROBE) reporting guideline for cross-sectional studies. This study was approved by the Massachusetts General Brigham Institutional Review Board and deemed exempt from informed consent because it was not considered human participants research.

### Data Sources

We queried the TRICARE website^26^ from November 16 to 20, 2020, to identify MTFs capable of providing emergency cesarean deliveries from all branches of service in the continental US. Capable MTFs were defined as those providing both obstetric and gynecologic services as well as emergency medical services. We then used their physical addresses to obtain geographic coordinates in Google Maps (Alphabet Inc). Data from the 2016 American Hospital Association annual survey^27^ were used to identify nonmilitary medical facilities capable of providing emergency cesarean delivery. We excluded Hawaii and Alaska because of the substantial reliance on air transportation for medical care in those states. We defined civilian hospitals capable of providing emergency cesarean deliveries as those that had clinical service lines for obstetric and emergency care, at least 1 operating room, and at least 1 surgical admission. Geographic coordinates for non-MTFs were also obtained from the 2016 American Hospital Association survey.^27^

We obtained data on health insurance coverage for TRICARE beneficiaries and their civilian dependents per county from the American Community Survey tables for 2017, which were available through ArcGIS Pro software, version 2.7 (Esri).^28^ Demographic characteristics of the general population were obtained from 202 key demographic indicators published by Esri.^29^ Age groupings for health insurance coverage categorized by data source did not allow us to ascertain the female population of childbearing age. We therefore aggregated female age groups (eg, 5-18 years, 19-34 years, and 34-64 years) to define the population of interest. Race and ethnicity were not examined because the data used for this study were aggregated and did not include further categorization by race or ethnicity.

### Outcomes

The primary goals for this study were to (1) identify MTFs within 30-minute catchment areas (defined as areas that were within a 30-minute travel time to a medical facility capable of providing emergency cesarean delivery care based on recommendations from the American College of Obstetricians and Gynecologists regarding timely emergent cesarean delivery)^30^ that were otherwise not served by civilian hospitals with emergency cesarean delivery capabilities; (2) estimate the proportion of female TRICARE beneficiaries who were dependent on MTF care for emergency cesarean deliveries (ie, no available civilian hospital within 30 minutes); and (3) estimate the proportion of the female civilian population who would gain access to emergency cesarean delivery services if MTFs in those important access areas were available to serve civilian populations. The secondary goal was to estimate the proportion of female TRICARE beneficiaries of childbearing age who did not have access to emergency cesarean delivery care within a 30-minute travel time.

### Statistical Analysis

We used ArcGIS Pro software, version 2.7 (Esri), to estimate population coverage and 30-minute travel times to facilities capable of providing cesarean delivery care. We used the service area layer of the network analysis tool to generate 30-minute drive-time polygons to facilities capable of providing cesarean delivery care. The network analysis tool measured all feasible driving routes to the defined destination (ie, the medical facility) and based drive-time estimates on posted speed limits and existing traffic control devices. We then used the enrich layer of the business analysis tool to calculate population coverages of interest within each service area, and we used data management tools to calculate summary statistics and estimate the population without coverage. We used SAS software, version 9.4 (SAS Institute Inc), and Excel software for Microsoft Office 365 (Microsoft Corp) to perform descriptive analyses and database management.

## Results

**Figure 1.**
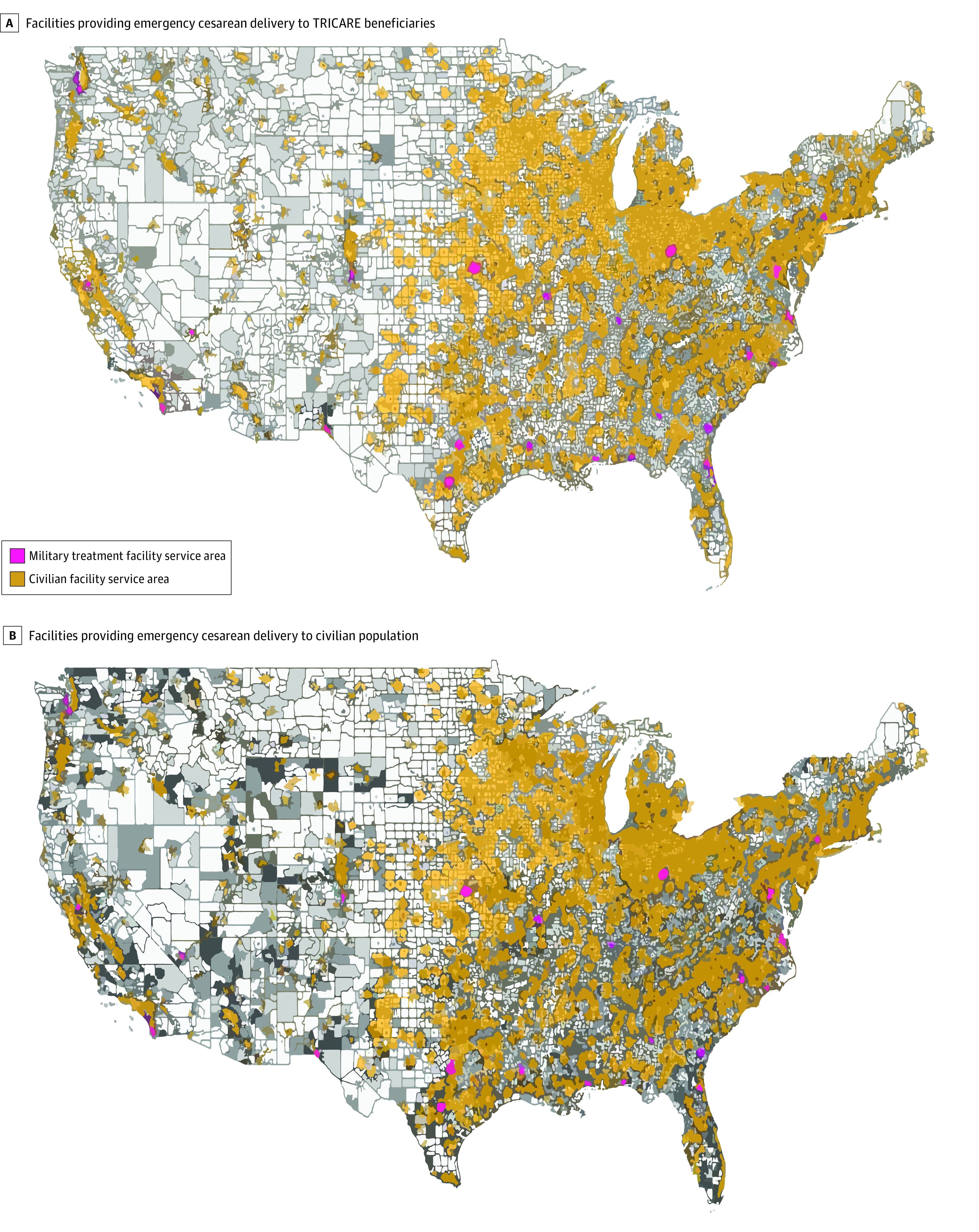
Coverage of Population Within 30-Minute Travel Time to Facilities Providing Emergency Cesarean Delivery Care A, Gray gradient reflects the population density of female TRICARE beneficiaries normalized by the total female population of TRICARE beneficiaries, with lighter gray representing lower density and darker gray representing higher density. B, Gray gradient reflects the population density of the female civilian population at the county level, with lighter gray representing lower density and darker gray representing higher density.

We identified 29 MTFs and 2363 civilian hospitals capable of providing emergency cesarean deliveries across the contiguous US. Overall, an estimated 167 759 762 women (3 640 000 TRICARE beneficiaries and 164 119 762 civilians) were included in these service regions. Population densities of TRICARE beneficiaries and civilians with respect to service areas are shown in Figure 1. Among 3 640 000 TRICARE beneficiaries, 1 775 207 (48.8%) had access to medical facilities providing emergency cesarean delivery care within a 30-minute travel time. Of those, 1 341 223 beneficiaries (36.8%) had access to care at civilian hospitals, and 433 984 beneficiaries (11.9%) had access to care that was only available at an MTF (Table 1). Among 164 119 762 civilians, 6 906 957 (4.2%) lived within a hypothetical 30-minute travel time to an MTF providing emergency cesarean delivery care.

**Table 1.** Travel Time Coverage to Civilian Hospitals and Military Medical Treatment Facilities Providing Emergency Cesarean Delivery Care

Coverage	No. (%)	
	Civilians	TRICARE beneficiaries
Total women, No.	164 119 762	3 640 000
Civilian hospital		
30-Min coverage	115 656 285 (70.5)	1 341 223 (36.8)
No coverage	48 463 477 (29.5)	2 298 777 (63.2)
Military treatment facility		
30-Min coverage	6 906 957 (4.2)	433 984 (11.9)
No coverage	157 212 805 (95.8)	3 206 016 (88.1)

**Figure 2.**
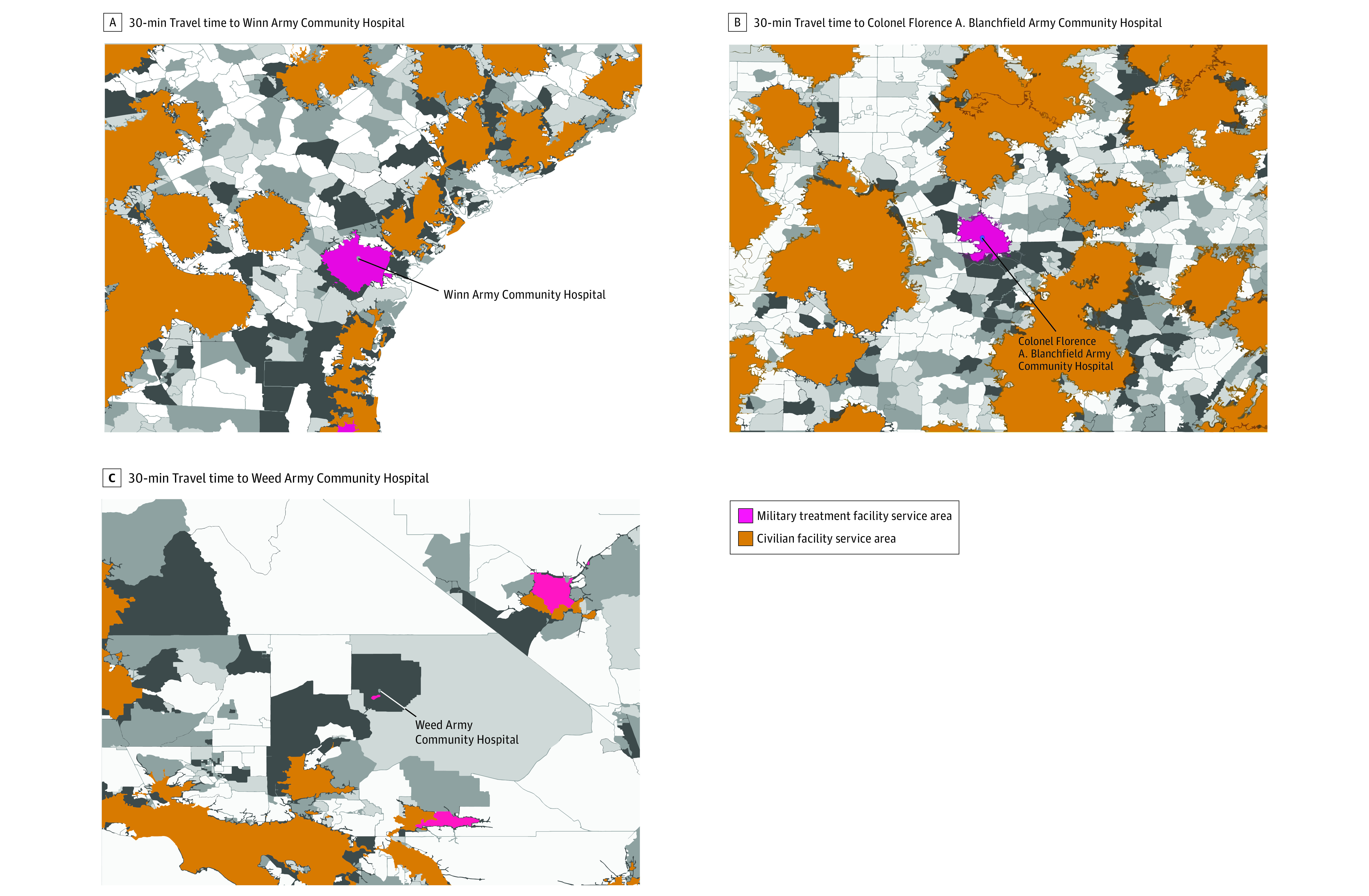
Sample of Service Areas for Military Treatment Facilities Providing Emergency Cesarean Delivery Care Yellow shading represents catchment areas for civilian hospitals. Gray gradient reflects population density of the female civilian population, with lighter gray representing lower density and darker gray representing higher density. A, Winn Army Community Hospital is located in Savannah, Georgia. A total of 18 022 female TRICARE beneficiaries and 60 781 potential female civilians were covered in the service area. B, Colonel Florence A. Blanchfield Army Community Hospital is located in Nashville, Tennessee. A total of 24 051 female TRICARE beneficiaries and 139 361 potential female civilians were covered in the service area. C, Weed Army Community Hospital is located in San Bernardino, California. A total of 18 804 female TRICARE beneficiaries and 83 307 potential female civilians were covered in the service area.

The 30-minute catchment areas of facilities capable of providing emergency cesarean delivery covered most of the northeastern, midwestern, and southeastern regions of the US, particularly urban areas (Figure 1). Substantial gaps in coverage were observed in the western region. We identified 17 of 29 MTFs (58.6%) capable of providing emergency cesarean delivery care that were located within 30-minute catchment areas. Three of those MTFs (Colonel Florence A. Blanchfield Army Community Hospital in Nashville, Tennessee; Weed Army Community Hospital in San Bernardino, California; and Winn Army Community Hospital in Savannah, Georgia) served as the only providers of emergency cesarean delivery care in their catchment areas. These MTFs covered approximately 28 440 TRICARE beneficiaries and had the potential to serve an additional 125 408 civilians within a 30-minute catchment area (Figure 2; Table 2). An additional 14 MTFs in 11 states (California, Colorado, Florida, Georgia, Louisiana, Mississippi, Missouri, Nevada, New York, North Carolina, and Washington) had catchment areas partially overlapping with civilian hospitals but also covered areas without alternative access to emergency cesarean delivery. These MTFs covered 158 768 TRICARE beneficiaries and had the potential to serve an additional 2 159 178 civilians within a 30-minute catchment area (Table 2).

**Table 2.** Military Medical Treatment Facilities Providing Emergency Cesarean Delivery Care

Medical treatment facility	Coverage, No.	
	Civilians	TRICARE beneficiaries
Total population covered by all MTFs and civilian hospitals	6 906 957	433 984
**Only MTF in 30-min catchment area providing emergency cesarean delivery**		
Winn Army Community Hospital, Georgia	38 889	7488
Colonel Florence A. Blanchfield Army Community Hospital, Tennessee	83 307	18 804
Weed Army Community Hospital, California	3212	2148
Subtotal	125 408	28 440
**MTF in 30-min catchment area partially overlapping with civilian hospitals^a^**		
Keller Army Community Hospital, New York	141 112	2219
Womack Army Medical Center, North Carolina	153 677	34 095
Martin Army Community Hospital, Georgia	73 772	8653
Naval Hospital Jacksonville, Florida	356 157	13 833
US Air Force Elgin Regional Hospital, Florida	58 558	12 174
US Air Force Medical Center Keesler, Mississippi	92 019	6281
General Leonard Wood Army Community Hospital, Missouri	20 372	5306
Bayne-Jones Army Community Hospital, Louisiana	21 749	4409
Evans US Army Community Hospital, Colorado	212 540	25 652
Mike O'Callaghan Federal Hospital, Nevada	607 723	10 322
Naval Hospital Bremerton, Washington	94 891	7507
Naval Hospital Camp Pendleton, California	78 657	7080
Robert E. Bush Naval Hospital, California	12 165	2312
Madigan Army Medical Center, Washington	235 786	18 925
Subtotal	2 159 178	158 768

Abbreviation: MTF, military treatment facility.

^a^ These facilities also covered areas without alternative access to emergency cesarean delivery care.

## Discussion

This cross-sectional study found that 58.6% of MTFs capable of providing emergency cesarean delivery were located in areas with the potential to improve access to emergency cesarean delivery care for civilians within a 30-minute travel time. These findings can be contextualized as follows: (1) these MTFs could be prioritized by the US Department of Defense, specifically when considering additional MTF reductions in access or scope of services during the ongoing MHS restructuring; and (2) these MTFs provide a distinct opportunity to explore additional military-civilian partnerships, which could increase access to emergency cesarean delivery care for TRICARE beneficiaries and underserved civilians living in rural areas.

In the MHS, federal regulations mandate that TRICARE beneficiaries have timely access to care and comprehensive obstetric coverage, including coverage for cesarean delivery.^8^ The MHS performs better than the average reported by the National Perinatal Information Center with regard to certain performance measures, such as the proportion of births via cesarean delivery (26% in the MHS vs 35% nationally), but the MHS has come under scrutiny for underperformance in measures such as managing shoulder dystocia, postpartum hemorrhage, and birth trauma or injury to the neonate.^8^ Despite the ability of the MHS to refer TRICARE beneficiaries to civilian hospitals when needed, many beneficiaries may have more limited access to specialty or subspecialty care, including maternal and fetal care.^7,11,31^ Identifying and prioritizing MTFs that may have strategic benefits for TRICARE beneficiaries and their families are important steps to achieving the obstetric care mandate^8^ while using finite resources efficiently.

In the US, the maternal mortality rate has increased over the past 10 years, from 15.7 pregnancy-associated deaths per 100 000 live births in 2006 to 16.9 pregnancy-associated deaths per 100 000 live births in 2016.^32^ In addition, infant mortality rates in the US are higher compared with rates in other high-income countries.^33^ As more rural hospitals close their obstetric units, women of childbearing age will likely experience increasingly limited access to obstetric care, including timely emergency cesarean deliveries.^34,35^ Given increasing maternal morbidity and mortality in the US, decreasing access to obstetric care in rural areas may further exacerbate maternal morbidity and mortality as well as health care disparities in rural areas. Thus, it is important to examine and consider resources in the US maternal health care system as a whole.^7^ The MHS has recently come under scrutiny for providing limited access to high-risk obstetric services and underperforming on certain performance outcomes, which has led the Government Accountability Office to request greater examination of civilian medical centers surrounding MTFs.^11^ Female civilians in the US have also experienced increases in severe maternal morbidity and mortality and decreases in access to obstetric care, prompting the American College of Obstetricians and Gynecologists to recommend improved regionalization of maternal care.^7^

These compounding situations provide an opportunity to explore additional military-civilian partnerships that may provide incremental benefits to both the MHS and the US population as a whole. Obstetric care is the largest service line in the MHS, and training military health care professionals in the management of obstetric emergencies is important to ensuring military readiness. The additional patient volume resulting from expanded access to obstetric care in MTFs may help to ensure the clinical readiness of military health care professionals. The military-civilian partnerships to address trauma and COVID-19 care at the Brooke Army Medical Center (through the Department of Defense Secretarial Designee Program and other special authorities) provide examples of the positive impact and benefit to both MHS and civilian patients.^18,22,23,36^ The 2017 National Defense Authorization Act^37^ contained provisions to facilitate such collaborations, including directives to provide treatment for selected civilians as a means of increasing clinical volume for military health care professionals, thereby ensuring they maintain clinical proficiency and combat readiness when working in noncombat settings.^37,38,39^

There has also been a call to expand access to MTFs to include Medicaid-eligible civilians in an effort to diversify the patient caseload of clinicians at MTFs and enable them to provide nontrauma care to civilian populations when deployed around the world.^40^ An early example of this expansion in access was the implementation of the Collaborative Efforts Statement, Multi-Federal Cancer Initiative,^41^ which allowed civilian patients with cancer who were receiving treatment at the National Institutes of Health Clinical Center to also receive care at the John P. Murtha Cancer Center at Walter Reed Army Medical Center in Bethesda, Maryland. Trauma care has set the precedent for successful partnerships, improving access to health care for underserved populations while providing a more diverse caseload for health care professionals at MTFs. However, there are many other facets of medical care in which partnerships can be developed, particularly maternal health care, which is a challenge for the nation.

### Limitations

This study has several limitations. These are primarily associated with the study’s ecological cross-sectional design. In addition, although no standard cutoff exists for travel times as a measure of timely access to care, we selected a threshold of 30 minutes as a proxy for emergency travel time to estimate and define clear catchment areas. Furthermore, the data sources did not allow for the ideal categorization of women of childbearing age, despite the fact that those older than childbearing age are susceptible to other gynecologic emergencies and would likely benefit from access to emergency care similar to that addressed in this study. Because our estimations were calculated at the population level, individual-level associations may differ in direction and extent from group-level associations (ie, the associations may be subject to the ecological fallacy, which occurs when group characteristics are applied to individuals).

## Conclusions

This cross-sectional study identified 17 MTFs that could improve access to high-quality cesarean delivery care for civilians in underserved regions of the US while also supporting military readiness. This enhanced access to cesarean delivery care, particularly in rural areas, has the potential to reduce inequities in the US health care system. Geospatial analyses provide an opportunity to strategically allocate limited resources based on population distribution. Such analyses can help inform policy makers and stakeholders about the need to prioritize important MTFs for continued services, identify areas in which military-civilian partnerships would be most beneficial, and identify where additional facilities are needed.
